# Fast and Accurate Background Reconstruction Using Background Bootstrapping

**DOI:** 10.3390/jimaging8010009

**Published:** 2022-01-11

**Authors:** Bruno Sauvalle, Arnaud de La Fortelle

**Affiliations:** Centre de Robotique, Mines ParisTech PSL University, 75006 Paris, France; arnaud.de_la_fortelle@mines-paristech.fr

**Keywords:** background reconstruction, background initialization, background generation, motion detection, background subtraction, scene parsing

## Abstract

The goal of background reconstruction is to recover the background image of a scene from a sequence of frames showing this scene cluttered by various moving objects. This task is fundamental in image analysis, and is generally the first step before more advanced processing, but difficult because there is no formal definition of what should be considered as background or foreground and the results may be severely impacted by various challenges such as illumination changes, intermittent object motions, highly cluttered scenes, etc. We propose in this paper a new iterative algorithm for background reconstruction, where the current estimate of the background is used to guess which image pixels are background pixels and a new background estimation is performed using those pixels only. We then show that the proposed algorithm, which uses stochastic gradient descent for improved regularization, is more accurate than the state of the art on the challenging SBMnet dataset, especially for short videos with low frame rates, and is also fast, reaching an average of 52 fps on this dataset when parameterized for maximal accuracy using acceleration with a graphics processing unit (GPU) and a Python implementation.

## 1. Introduction

We consider in this paper the task of static background reconstruction: starting from a sequence of images $X=X_{1},\ldots ,X_{N}$ of a scene showing moving objects, for example cars, bikes or pedestrians, the goal is to recover the image of the background of this scene, without any of the moving objects. This task is fundamental in image analysis: The moving objects appearing in the scene may be considered as a nuisance, and background reconstruction allows to remove them completely and focus on the analysis of the background, for example to localize or map the scene. More frequently, for example for video surveillance or traffic monitoring, the moving objects are the main object of interest and the background itself is considered as a nuisance, so that background reconstruction is a first step which can be used to extract and analyze the moving objects of the scene. The task of background reconstruction should not be confused with the task of background modeling that involves building a statistical model of the background images whereas the task of background reconstruction requires to predict a unique background image.

It is often assumed that all the images $X_{1},\ldots ,X_{N}$ share the same background, which is then called a static background. In this case, the output of the algorithm is composed of only one background image $\hat{X}$. It is however also possible that the backgrounds are slightly different in each image, for example if the illumination conditions change or if the camera is moving. In this situation, we expect a background reconstruction algorithm to output a sequence of backgrounds ${\hat{X}}_{1},\ldots ,{\hat{X}}_{N}$ and we say that the background reconstruction is dynamic. In this paper, we consider the problem of static background reconstruction.

This problem is a difficult because there is no formal definition of what should be considered as background or foreground. Moving trees, fountains and moving shadows are examples of instances that are usually considered as belonging to the background although they show moving features. Other challenges such as illumination changes or the presence of objects staying still for a short time (a problem called intermittent motion) may severely impact the quality of a background reconstruction model.

One should distinguish between online methods, where the length of the dataset is unknown and the background reconstruction algorithm has to update the background model in real-time and batch methods, where the algorithm is provided with a fixed dataset. The method proposed in this paper is a batch method.

The main contribution of this paper are the followings:

- We implement a new consistency criterion for background estimation: The background estimate produced by a background estimation method should not change if we perform the background estimation using only pixels that are considered as background pixels with regards to this background estimate.
- We then show that this consistency criterion can be described as an optimization criterion and that that the associated optimization problem can be efficiently solved using stochastic gradient descent.

The paper is organized as follows: In Section 2, we review related work in static background reconstruction. In Section 3, we describe the proposed algorithm. Experimental results are then provided in Section 4.

## 2. Related Work

Temporal median filtering (TMF) [1] simply computes the background color for a pixel *p* as the median of the colors of this pixel on all the images $X_{1},\ldots ,X_{N}$. Despite its simplicity, this algorithm and its variant temporal median filter with gaussian filtering (TMFG) [2] perform very well on several scene categories.

The current state-of-the-art models for unsupervised fixed background reconstruction are the Superpixel motion detection algorithm (SPMD) [3] and LaBGen-OF [4]. Both of these models, as well as the frame selection method and efficient background estimation procedure (FSBE) [5], implement the idea that the regions of the input frames showing foreground objects should not be considered to compute the background.

SPMD first selects the longest sequence with stable illumination, then uses superpixel segmentation [6], and removes all superpixels with contain at least one moving pixel using a frame difference method to detect moving pixels. The various pixel values associated with one pixel position are then clustered, and the median value of the best cluster is selected to produce the background value. Removing superpixels associated with moving pixels for background initialization is also developed in [7].

LaBGen and LaBGen-P [8,9] assume that a background/foreground segmentation algorithm is available. For a given pixel or spatial patch, these models select the frames showing the lowest number of foreground pixels, and then perform a pixel-wise median filtering. LaBGen-OF is a variant which uses an optical flow algorithm [4]. LaBGen-semantic is another variant with uses a supervised semantic segmentation model [10]. This model has also been adapted in [11] to detect illumination changes and use only a subsequence with stable illumination conditions.

The FSBE algorithm (frame selection and background estimation) [5] assumes that an optical flow algorithm is available. It first selects a sequence of frames where the illumination conditions do not change too much. Using the optical flow algorithm, it classifies as background all pixels which have an optical flow magnitude below some threshold and corrects this classification if it detects high dynamic motion or foreground intermittent motion in the sequence. It then takes the pixel-wise average of the selected background pixels.

Instead of only removing pixels or patches which show moving objects before performing temporal median filtering, some models [12,13] try to select for each pixel only one patch from the various frames, which is considered to be the best candidate to represent the background, so that temporal median filtering is not needed. Photomontage [14] builds the background as a seamless montage composed of patches extracted from the images $X_{1},\ldots ,X_{N}$ so that the likelihood of the color at each pixel is maximum with respect to the probability distribution function formed from the color histogram of all pixels in the span.

Some models try to benefit from the fact that if the content of the background is known in some part of the image, it is easier to distinguish between background and foreground objects in adjacent parts of the image, using a spatial or temporal consistency criterion. The neighborhood exploration based background initialization (NExBI) algorithm [15] divides the frame in blocks, and perform a temporal clustering for each block location. A preliminary partial background model is then created for the blocks that remain stable during all the sequence, i.e., where all the image patches associated with this block form only one cluster, and then iteratively extended to the whole image as a puzzle game by enforcing consistency between candidate background blocks and the partial background model. Other iterative block completion models have been proposed in [16,17,18,19,20,21].

Another approach which has been investigated for background reconstruction is to consider the sequence $X=X_{1},\ldots ,X_{n}$ as a 3D tensor or a spatiotemporal matrix and to decompose it as the sum of a low-rank part, which is assumed to be representative of the background, and a sparse part, which should be representative of the foreground objects. For example, the Motion-assisted spatiotemporal clustering of low-rank algorithm (MSCL) [22], which is a dynamic background reconstruction model using robust principal component analysis (RPCA) [23], is able to obtain better results than state of the art fixed background reconstruction models on the scene background modeling (SBMnet) dataset using this method, although it is not directly comparable to those models because it requires some human supervision to select the final frame $\hat{X}$ from the various predicted backgrounds ${\hat{X}}_{1},\ldots ,{\hat{X}}_{n}$ associated with the frames $X_{1},\ldots ,X_{n}$. Another linear method proposed to extract a low rank background is to apply singular value decomposition (SVD) to spatiotemporal slices of the tensor X, consider that the first principal subspace is associated with the background, and use the other components to detect foreground objects, which can then be excluded from the background computation [24,25].

The background estimation by weightless neural network (BEWIS) [26] and self-organizing background subtraction (SOBS) algorithms [27,28,29] involve weightless neural networks, which are used as containers to build a statistical model of the background.

The current top performing algorithms for background reconstruction do not use deep learning techniques, but several papers have proposed to use them for fixed background reconstruction:

Fully-concatenated Flownet (FC-Flownet) [30] is a convolutional network with an architecture similar to a U-net which is used to predict a background from a set of 20 color images in a single inference step. Due to memory restrictions, the images are cut in superposed 64 × 64 patches, and the 20 patches associated with one location are given as input to the convolutional network. The output patches are then aggregated to build the background. The network is trained end-to-end using samples and ground truths coming from 54 different sequences.

Background modeling Unet (BM-Unet) [31] is a background reconstruction model which also uses a U-net network but is trained without any supervision or ground-truth data and can perform both fixed and dynamic background reconstruction. For fixed background reconstruction, it is trained with pairs of random images sampled from one frame sequence. Using the first image, the U-net network predicts a probability distribution over the possible 256 values of each pixel of the output image, and the second image is used as a target.

Deep context prediction (DCP) [32] considers the background reconstruction problem as an inpainting problem: Using an optical flow algorithm, it first computes the motion mask associated with the current frame and removes from this frame the pixels associated with this motion mask. It then uses a multi-scale neural path synthesis network [33] to fill the holes in the image and obtain a clean background. Other data reconstruction methods using classical matrix completion or exemplar-based approaches are also possible [34,35,36].

We refer to available surveys [37,38] for a more detailed description of related work.

## 3. Proposed Algorithm for Background Reconstruction

### 3.1. Motivation

We have noted in the review of previous work the good results of temporal median filtering, despite its simplicity, and observe that the two best unsupervised algorithms for background reconstruction, SPMD and LabGen-OF, also use some form of temporal median filtering. One can intuitively understand that background reconstruction involves performing some form of averaging of the input frames, and that computing the median will give better results than computing the average of the frames because the median is more robust to outliers.

We note however that using median filtering on color images may lead to inconsistencies. Let us for example consider RGB images showing a red background with large green and blue foreground objects. Assume that in the sequence considered, each red background pixel is masked by a green object during 26% of sequence duration and by a blue object during another 26% of the sequence duration. The red color channel of any pixel will then be equal to zero during 52% of the sequence, and the blue and green channels are also equal to zero during 74% of the sequence. As a consequence, the result of median filtering on such a sequence is a uniform black image, which is clearly not satisfactory.

One can think that a better method to select the background color of an image from a frame sequence would be first to guess in each frame which pixels are background pixels and then to consider only those pixels for temporal median filtering. However, to be able to guess which pixels are background pixels, we need to have some estimate of the background. The main idea introduced in this paper is that we can successfully build an iterative optimization process for background reconstruction, using the current estimate of the background to guess which pixels are background pixels and then refining the estimate of the background by performing temporal median filtering on those pixels only.

### 3.2. Bootstrap Weights

We observe that temporal median filtering can be described as a minimization problem associated with a $L_{1}$ error loss. More precisely, for a sequence of color images $X_{1},\ldots ,X_{N}$ of size h×w, noting $x_{n,c,i,j}$ the value (normalized in the range [0,1]) of the pixel associated with the image $X_{n}$ and the color channel *c* at position (i,j) with 1≤i≤h and 1≤j≤w, the $L_{1}$ error loss associated with some background reconstruction $\hat{X}$ can be described as

(1) $$L_{1}\left(\hat{X},{\left(X_{n}\right)}_{1\leq n\leq N}\right)=\frac{1}{N}\sum_{n=1}^{N}L_{1}\left(\hat{X},X_{n}\right)$$

with

(2) $$L_{1}\left(\hat{X},X_{n}\right)=\frac{1}{hw}\sum_{i=1,j=1}^{h,w}\sum_{c=1}^{3}\left|{\hat{x}}_{c,i,j}-x_{n,c,i,j}\right|,$$

and it is immediate that if we take each ${\hat{x}}_{c,i,j}$ to be a median of the sequence ${\left(x_{n,c,i,j}\right)}_{1\leq n\leq N}$, then we obtain a minimum of this loss function, considering that the derivative of $\left|{\hat{x}}_{c,i,j}-x_{n,c,i,j}\right|$ with respect to ${\hat{x}}_{c,i,j}$ is equal to 1 if ${\hat{x}}_{c,i,j}-x_{n,c,i,j}>0$ and −1 if ${\hat{x}}_{c,i,j}-x_{n,c,i,j}<0$.

**Figure 1:**
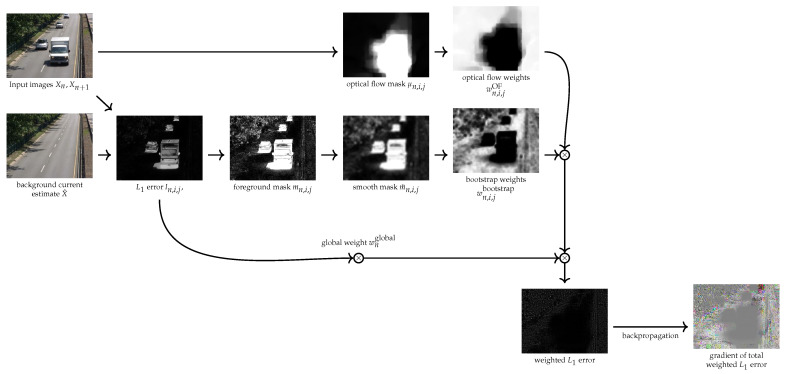
Schematic of loss function and gradient computation (Images are normalized in the range [0,1]).

We bootstrap the current estimate of the background to smoothly restrict this loss function to background pixels only. We then give a low weight, called a bootstrap weight, to the pixel-wise error terms $\sum_{c=1}^{3}\left|{\hat{x}}_{c,i,j}-x_{n,c,i,j}\right|$ associated with foreground pixels in the loss function. These bootstrap weights are computed in the following way (Figure 1):

Let us note $l_{n,i,j}$ the sum of the $L_{1}$ errors for each color at the pixel (i,j) between the predicted image $\hat{X}$ and the input image $X_{n}$ for all the color channels:

(3) $$l_{n,i,j}=\sum_{c=1}^{3}\left|{\hat{x}}_{c,i,j}-x_{n,c,i,j}\right|$$

If at least one of the color channels give a high error, then $l_{n,i,j}$ is large and we will consider that the pixel (i,j) of the image $X_{n}$ is a foreground pixel. We then build a soft foreground mask $m_{n}\in {\left[0,1\right]}^{h\times w}$ for the image $X_{n}$ using the formula

(4) $$m_{n,i,j}=\tanh\left(\frac{l_{n,i,j}}{\tau_{1}}\right)$$

where $\tau_{1}$ is some positive hyperparameter, which can be considered as a soft threshold. As a consequence, $m_{n,i,j}$ is close to zero for values of $l_{n,i,j}$ close to zero (background pixels), and close to 1 for values of $l_{n,i,j}$ which are significantly larger than $\tau_{1}$ (foreground pixels).

This mask will however be noisy. We then compute a spatially smoothed version ${\tilde{m}}_{n,i,j}$ of this mask by averaging using a square kernel of size (2k+1)×(2k+1), with $k=\left\lfloor w/r\right\rfloor$ (where *w* is the image width and *r* is some integer hyperparameter):

(5) $${\tilde{m}}_{n,i,j}\left(\hat{X},X_{n}\right)=\frac{1}{{\left(2k+1\right)}^{2}}\sum_{l=-k,p=-k}^{l=k,p=k}m_{n,i+l,j+p}$$

We then finally define the associated pixel-wise bootstrap weights $w_{n,i,j}^{\mathrm{bootstrap}}$ as

(6) $$w_{n,i,j}^{\mathrm{bootstrap}}=e^{-\beta {\tilde{m}}_{n,i,j}},$$

where β is some positive hyperparameter, which we call the bootstrap coefficient.

For pixels which are considered to be background pixels (${\tilde{m}}_{n,i,j}≃0$), this weight will be close to 1 and will not change the pixel-wise loss terms $\sum_{c=1}^{3}\left|{\hat{x}}_{c,i,j}-x_{n,c,i,j}\right|$ associated with these pixels. However for pixels which are considered as foreground pixels (${\tilde{m}}_{n,i,j}≃1$), this weight will have a very low value close to $e^{-\beta }$, which means that the associated pixel-wise loss terms will get a very low importance in the loss function.

### 3.3. Optical Flow Weights

We have seen that background reconstruction algorithms could be improved by using informations provided by optical flow models to remove parts of an image showing moving objects. We use the same approach to improve the loss functions $L_{1}$. We then define optical flow weights associated with each pixel $x_{n,i,j}$ which will be close to zero if this pixel appears to be a moving pixel and has to be removed from the loss function computation. These weights are computed in the following way (cf Figure 1):

We use an external algorithm (OpenCV implementation of Dense Inverse Search algorithm [39]) to obtain an estimate of the magnitude $\phi_{n,i,j}$ of the optical flow associated with each pixel (i,j) of an image $X_{n}$. We chose this algorithm because it is very fast compared to other available optical flow implementations. We first normalize $\phi_{n,i,j}$ with respect to the image width *w* and then define an optical flow mask $\mu_{n,i,j}$ using the formula

(7) $$\mu_{n,i,j}=\min\left(1,\frac{\phi_{n,i,j}}{w\tau_{2}}\right),$$

where the hyperparameter $\tau_{2}$ can also be considered as a threshold. This mask is then equal to 1 for high values of the optical flow $\phi_{n,i,j}$, which suggests that the associated pixels show a moving object, and it is close to zero if no motion is detected by the optical flow algorithm at the associated pixel.

The weight associated with this optical flow mask is then defined as

(8) $$w_{n,i,j}^{\mathrm{OF}}=e^{-\phi \mu_{n,i,j}},$$

where ϕ is another positive hyperparameter. This weight will then be equal to 1 if no motion is detected at the associated pixel, and close to $e^{-\phi }$ if a significant motion is detected, which suggests that the associated pixel is not a background pixel. $w_{n,i,j}^{\mathrm{OF}}$ is, however, set to 1 for all pixels on short videos (less than ten images), considering that optical flows computed from sequences with very low frame rates are not reliable.

### 3.4. Abnormal Image Weights

If the number of images in the dataset is large, we can afford to give a low weight to images which appear to be abnormal, for example if the illumination conditions are different on these images compared to the predicted background, or if there are too many pixel errors on the image. We then first compute the average $L_{1}$ error ${\bar{l}}_{n}$ of the image $X_{n}$ as

(9) $${\bar{l}}_{n}=\frac{1}{hw}\sum_{i,j}l_{n,i,j}$$

and define a global weight associated with each image $X_{n}$ as

(10) $$w_{n}^{\mathrm{global}}=e^{-\gamma {\bar{l}}_{n}},$$

where γ is another positive hyperparameter. As a consequence, this weight $w_{n}^{\mathrm{global}}$ will be close to zero if the image $X_{n}$ is globally very different from the current estimate $\hat{X}$ of the background. We use this global weight if the size of the dataset is greater than 10. It should be noted that this weight is not pixel-specific, as opposed to the bootstrap weights and optical flow weights, but is assigned to a complete frame.

### 3.5. Management of Intermittent Motion

Existing benchmarks for background reconstruction require that objects which remain still for a long time in the sequence be considered as foreground objects if they are moving during some part of the sequence. This challenge is very difficult and is not addressed by the previous weights. In order to handle it, we follow Javed et al. [22,40] and remove from the frames sequence all frames which are not showing any motion. More precisely, we first compute the maximum $\mu_{n}^{*}$ of the optical flow mask values $\mu_{n,i,j}$ of the image $X_{n}$ as defined in previous section, and remove this image if $\mu_{n}^{*}<\tau_{3}$, where $\tau_{3}$ is another threshold hyperparameter. The motivation of this suppression is that it appears that images containing still foreground objects are often motionless images, so that removing them improves the robustness of the proposed model against the intermittent motion challenge. We apply this motionless frame suppression when the number of frames in the sequence is higher than 10, considering as in previous section, that removing frames when the number of frames is very low will impact negatively the quality of the results. We note $N^{\prime }\leq N$ the number of frames after motionless frame suppression.

### 3.6. Statement of the Optimization Problem

Finally, the loss function is adapted using these weights and becomes the following:

(11) $$L_{W}\left(\hat{X},{\left(X_{n}\right)}_{1\leq n\leq N^{\prime }}\right)=\frac{1}{N^{\prime }hw}\sum_{n=1,i=1,j=1}^{N^{\prime },h,w}w_{n}^{\mathrm{global}}w_{n,i,j}^{\mathrm{bootstrap}}w_{n,i,j}^{OF}\sum_{c=1}^{3}\left|{\hat{x}}_{c,i,j}-x_{n,c,i,j}\right|$$

We are then interested to solve the following optimization problem: *Considering the dataset ${\left(X_{n}\right)}_{1\leq n\leq N^{\prime }}$, find an image $\hat{X}$ so that, when the weights $w_{n}^{\mathrm{global}}$ and $w_{n,i,j}^{\mathrm{bootstrap}}$ are considered as constants, the loss function $L_{W}\left(\hat{X},{\left(X_{n}\right)}_{1\leq n\leq N^{\prime }}\right)$ is minimal with respect to $\hat{X}$*.

**Figure 2:**
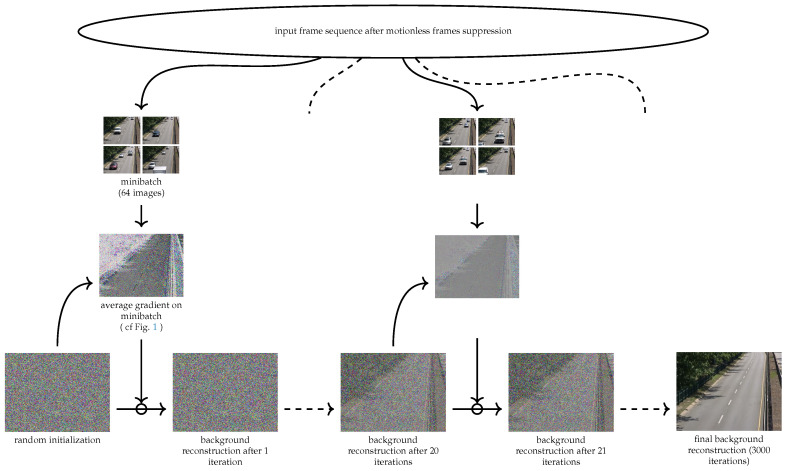
Overview of the stochastic gradient descent optimization process.

We can find a solution to this problem by performing an iterative computation of the weighted median of the images using the various weights defined in the previous paragraph followed by an update of the weights. We observe, however, that the images produced using this method are not smooth and that additional regularization is necessary. We then propose to use stochastic gradient descent on the loss function $L_{W}\left(\hat{X},{\left(X_{n}\right)}_{1\leq n\leq N^{\prime }}\right)$ using standard deep learning tools. The pixel values ${\hat{x}}_{c,i,j}$ are then considered as parameters and optimized using stochastic gradient descent (Figure 2).

It should be noted that performing a stochastic gradient descent on this loss function is not equivalent to minimizing it: During the optimization process, the weights $w_{n,i,j}^{\mathrm{boostrap}}$ and $w_{n}^{\mathrm{global}}$ depend on the current estimation of the background and change; we then call these weights dynamic weights. At each iteration they are, however, considered as fixed so that we do not compute and use the gradient of the loss function with respect to the value of these weights.

## 4. Evaluation of the Proposed Model

Two public benchmarks are available for the evaluation of fixed background reconstruction models: the SBMnet dataset [41] and the SBI dataset [42]. We first provide a quantitative evaluation of the proposed model on those two datasets. We then perform an ablation study and some computation speed measurements.

### 4.1. Implementation Details

A desktop computer with an Intel Core i7 7700K@4.2GHz CPU and a Nvidia RTX 2080 TI GPU is used for this experiment.The model is implemented in Python using the Pytorch framework and is publicly available on the Github platform. We use the Adam optimizer [43], with learning rate 0.03 and batch size 64, reduced by a factor of 10 when 3/4 of the epochs have been computed. The number of epochs depends on the size of the dataset and is adjusted so that the total number of optimization iterations is close to 3000, with a minimum of two epochs. In order to accelerate computations, each frame sequence is fully loaded in the GPU video RAM during the optimization process. A manual hyperparameter search has been performed using the video sequences of the SBI and SBM datasets for which a ground truth is available. The hyperparameters have then been set to the following values: $\beta =6,\phi =2,\gamma =3,r=75,\tau_{1}=0.25,\tau_{2}=255/40000,\tau_{3}=240/255$. Before starting the optimization, background image pixel color values are initialized with random numbers sampled from a uniform distribution between 0 and 1. The DIS optical flow OpenCV implementation is used with the FAST preset mode. In order to obtain a low gradient when $l_{n,i,j}$ is close to zero, we replace the expression $\left|{\hat{x}}_{c,i,j}-x_{n,c,i,j}\right|$ with a smooth $L_{1}$ loss using a threshold equal to 3 (assuming the pixel values are scaled in the range 0–255). When $\left|{\hat{x}}_{c,i,j}-x_{n,c,i,j}\right|$ is lower than 3, we replace it with the quadratic expression $0.5{\left({\hat{x}}_{c,i,j}-x_{n,c,i,j}\right)}^{2}/3$, otherwise we replace it with $|{\hat{x}}_{c,i,j}-x_{n,c,i,j}|-0.5\times 3$.

### 4.2. Evaluation on SBMnet dataset

The SBMnet dataset [41] (http://scenebackgroundmodeling.net/, accessed on 20 November 2021) is composed of 79 sequences, which have been selected to cover a wide range of challenges and are representative of typical indoor and outdoor visual data captured today in surveillance, smart environment, and video database scenarios. The dataset includes the following eight categories with associated challenges: basic, intermittent motion, clutter, jitter, illumination changes, background motion, very long and very short. Although this dataset is freely available on the SBMnet website, ground truth images are publicly available for only 18 frame sequences, either on the SBMnet website or on the SBI dataset website. In order to benchmark a new algorithm, one has to submit the predicted fixed background images associated with each frame sequence to the website, which performs the evaluation of the submitted results.

Six criteria are computed to evaluate the accuracy of background reconstruction:

- Average Gray-level Error (AGE);
- Percentage of Error Pixels (pEPs);
- Percentage of Clustered Error Pixels (pCEPs);
- Multi-Scale Structural Similarity Index (MS-SSIM);
- Peak-Signal-to-Noise-Ratio (PSNR);
- Color image Quality Measure (CQM).

We refer to [41] for the full definition of these criteria. A good background reconstruction should minimize the criteria AGE, pEPs and pCEPs, but maximize the criteria MS-SSIM, PSNR and CQM. We have computed the 79 background images using the proposed algorithm and uploaded the reconstructed backgrounds to the SBMnet website, which provided the evaluation results described in Table 1 and Table 2.

We provide a comparison of the proposed model with models that are fully unsupervised, i.e., which do not use a supervised segmentation model (such as LabGen-semantic) and do not require any human supervision. The proposed model, named BB-SGD (background bootstrapping using stochastic gradient descent) obtains a better average score than all referenced unsupervised models on all criteria as shown in Table 1. Table 2 lists AGE results per category of the SBMnet dataset. It shows that the proposed models shows better AGE results than all referenced unsupervised models on 4 categories: basic, clutter, background motion and very short video, with a 15% accuracy improvement on the very short video category compared to the best unsupervised model in this category, which illustrates the efficiency of the bootstrapping mechanism introduced in the proposed model considering that for these sequences, the optical flow weights and global weights are not used and no frame is suppressed.

### 4.3. Evaluation on SBI Dataset

The SBI dataset [42] (https://sbmi2015.na.icar.cnr.it/SBIdataset.html, accessed on 20 November 2021) is composed of 14 image sequences. Ground truth backgrounds are available for all sequences. We use the Matlab tool available on the SBI website for fair comparison with other models, but do not report the CQM results considering that other sequences were evaluated with a Matlab tool which included a bug for the CQM computation, as indicated in the SBI website. We run the proposed model on the SBI dataset using the same hyperparameters as those used for the SBMnet dataset. The results of this evaluation are listed in Table 3 and show that the proposed model obtains better results than all other compared unsupervised models for the evaluation criteria AGE, MS-SSIM and PSNR, and is ranked second for the criteria pEPs and pCEPs.

### 4.4. Ablation Study

In order to check the contribution of the various weights described in this paper, we provide results obtained using truncated versions of the proposed model while keeping the hyperparameters fixed: Version 0 does not use any weight and does not remove motionless frames, and is then equivalent to temporal median filtering. Version 1 uses only the optical flow weights and does not remove motionless frames. Version 2 uses both optical flow weights and global weights and does not remove motionless frames. Version 3 uses bootsrap weights, global weights and optical flow weights, but does not remove motionless frames. The AGE scores obtained by these truncated models on the 18 videos of the SBMnet dataset for which a ground truth is available and using the evaluation tool available on the SBMnet website are provided in Table 4. They show that temporal median filtering (v0) gives the best results for five scenes, confirming that this is a good baseline. Introducing optical flow weights (v1) improves average AGE scores on scenes of the “clutter” category, but has no beneficial impact on other categories. Adding global weights (v2) has a positive impact on the “illumination change” category, which was expected, but also on the “clutter” category”. Adding bootstrap weights has an impact on the “clutter” category, but also on the “short video” category. Finally, removing motionless frames, which leads to the full model, has a positive impact on the “intermittent motion” category, which was expected, but also on the scene “boulevardJam” of the “clutter” category, which also shows some intermittent motions.

### 4.5. Computation Time

We have performed computation times measurements and tested the impact of reducing the number of optimization iterations, while keeping all other parameters frozen, excluding the learning rate. The results of these experiments are provided in Table 5. The total computation times necessary to reconstruct the 79 backgrounds from the associated video sequences of the SBMnet dataset are estimated by performing a sequential computation for all the videos, so that the computation times indicated in this table are the sum of the computation times of each of the 79 videos. If we divide the number of frames of the full dataset (73,355) with the total computation time of the proposed model, which is 1409 s, we obtain an average of 52 frames per second (fps). Table 5 shows, however, that the number of optimization iterations can be reduced from 3000 to 250, increasing the average speed to 187 fps, without major impact on the overall accuracy of the algorithm. The computation times with such a low number of iterations are mainly associated with optical flow computations and JPEG images decoding.

Although the proposed model requires a GPU, these computation time measurements compare very favorably with the processing speeds reported by the authors of other models. The average computation speed of LabGen-OF is estimated to 5 fps in [4]. The computation speed of SPMD is estimated in [3] to 1.6 fps for 640 × 480 images and 22.8 fps for 200 × 144 images using a Intel Core i7 2600@3.4Ghz CPU.

The asymptotic time complexity of the proposed algorithm is $O(p^{2})$ where p=hw is the number of pixels of an image. It does not depend on the number of frames of the input frame sequence since a maximum of 64×3000 images are sampled from the input sequence (3000 minibatches of 64 images) and optical flow computations can be restricted to those images only. The quadratic expression $O(p^{2})$ is a consequence of Equation (5), which involves a kernel which has a size proportional to the size of the images for *r* fixed.

### 4.6. Image Samples

**Figure 3:**
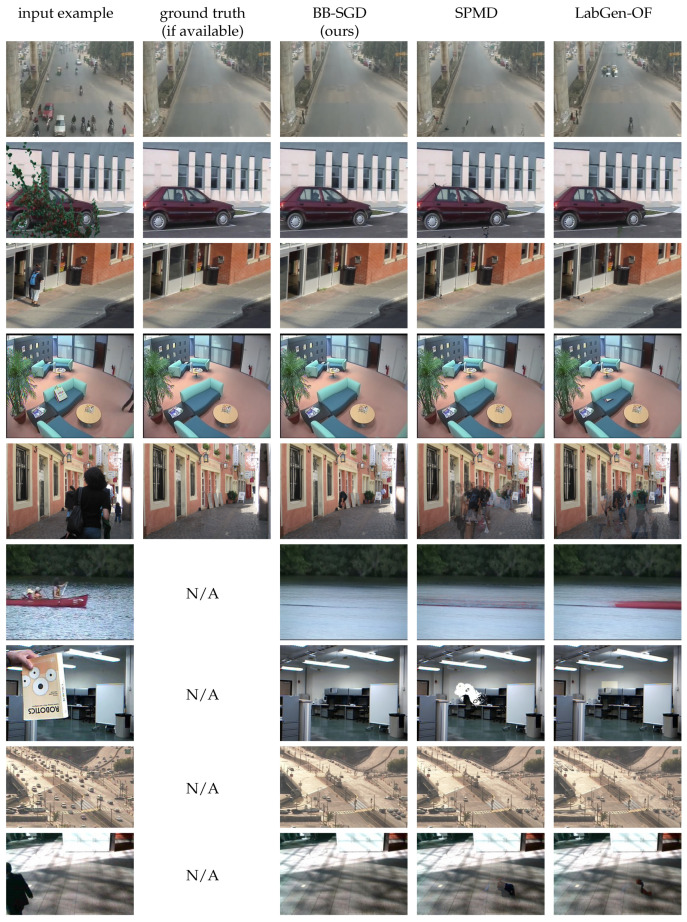
Examples of background reconstruction using the proposed model and comparison with SPMD and LabGen-OF.

**Figure 4:**
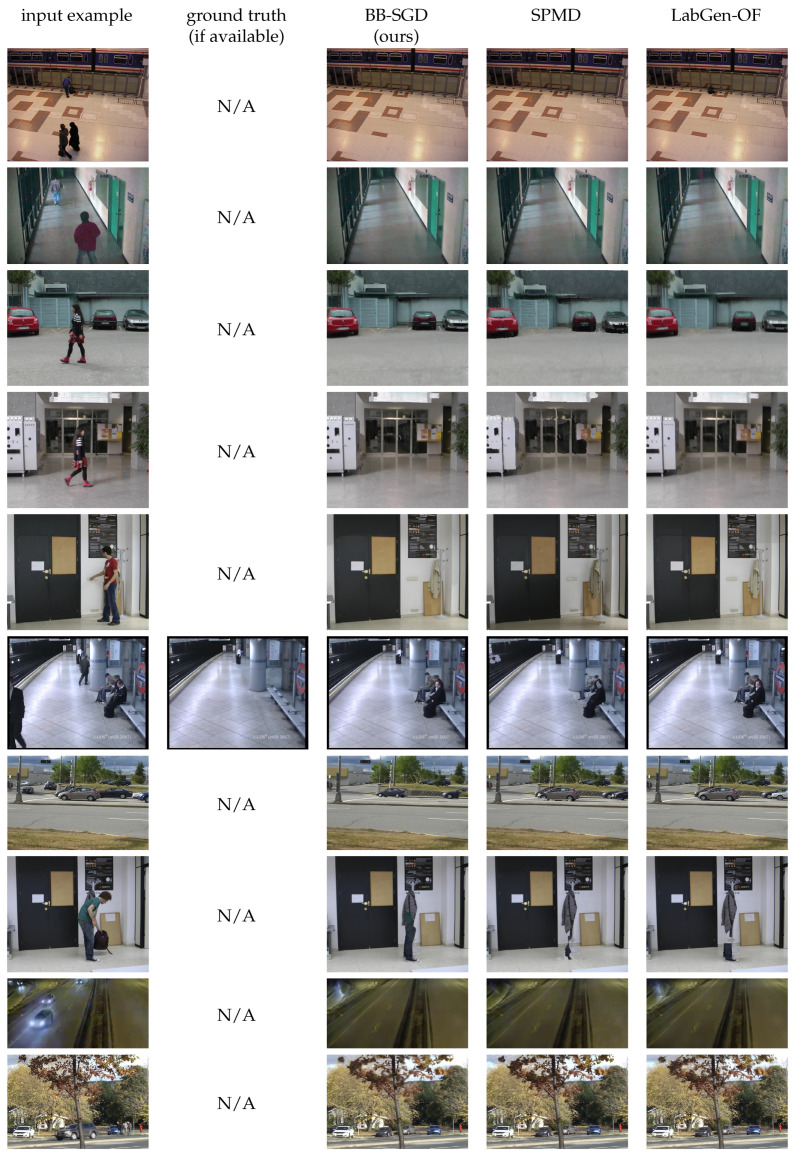
Examples of background reconstruction. The bottom five rows show examples of low quality reconstructions.

Figure 3 and Figure 4 show for qualitative evaluation some examples of background reconstruction for sequences of the SBMnet dataset, with the associated ground-truth when it is available and a comparison with the results obtained with LabGen-OF and SPMD. The bottom five rows of Figure 4 show some examples of poor quality reconstructions suffering from challenging issues such as intermittent motion, headlights and moving trees.

### 4.7. Hyperparameter Tuning

The proposed model involves a significant number of hyperparameters. Although the default hyperparameters proposed in Section 4 allow us to obtain state-of-the-art performances on existing benchmarks, these hyperparameters may be fine-tuned to improve results on specific situations or use cases. We provide below some indications on the influence of the main hyperparameters:

- $\tau_{1}$: the soft threshold used for computing soft foreground mask should be decreased for frame sequences with very low average illumination.
- $\tau_{2}$: the soft threshold used for computing optical flow masks should be decreased for video sequences with high frame rates and increased for sequences with low frame rates, considering that optical flow values are lower for a high frame rate sequences and higher for a low frame rate sequences.
- Optical flow weight ϕ: as shown in the ablation study, the use of optical flow weights is only necessary for highly occluded scenes. More precise results may be obtained by setting this parameter to lower value if a high level of occlusion is not expected.
- *r*: the value of *r* is associated with the expected sizes of the foreground objects: If it is forecast that the scenes will contain only small foreground objects, this value may be increased on high definition images for faster training.
- Bootstrap coefficient β: a lower value of β leads to faster training, but decreases the ability to handle occlusions. A higher value of β may lead to slower or unstable training and artifacts in the final image.
- Global weight γ: increasing the value of γ may be useful to handle low intensity illumination changes.

## 5. Conclusions

We have presented a new algorithm for fixed background reconstruction using stochastic gradient descent which is simple, fast using a GPU and is more accurate than the current state of the art. This shows that with modern hardware, stochastic gradient descent can be used efficiently for real-time applications and that the tools and frameworks which have been recently developed for deep learning and neural networks can also be useful for other optimization problems with a proper design of the loss function. Further works include using the same approach to handle the task of dynamic background reconstruction and change detection.

## Figures and Tables

**Table 1:** Evaluation results per criteria on the SBMnet 2016 dataset. ↓ indicates lower score is better, ↑ indicates higher score is better. Source: SBMnet website http://pione.dinf.usherbrooke.ca/results/294/ (accessed on 20 November 2021).

Method	Average AGE ↓	Average pEPs↓	Average pCPEPs↓	Average MS-SSIM↑	Average PSNR↑	Average CQM↑
BB-SGD (ours)	**5.6266**	**0.0447**	**0.0147**	**0.9478**	**30.4016**	**31.2420**
SPMD [3]	6.0985	0.0487	0.0154	0.9412	29.8439	30.6499
LabGen-OF [4]	6.1897	0.0566	0.0232	0.9412	29.8957	30.7006
FSBE [5]	6.6204	0.0605	0.0217	0.9373	29.3378	30.1777
BEWIS [26]	6.7094	0.0592	0.0266	0.9282	28.7728	29.6342
NExBI [15]	6.7778	0.0671	0.0227	0.9196	27.9944	28.8810
Photomontage [14]	7.1950	0.0686	0.0257	0.9189	28.0113	28.8719
SOBS [28]	7.5183	0.0711	0.0242	0.9160	27.6533	28.5601
Temporal Median Filter [1]	8.2761	0.0984	0.0546	0.9130	27.5364	28.4434

**Table 2:** Evaluation results for the AGE criterion per category on the SBMnet 2016 dataset. Source: SBMnet website http://pione.dinf.usherbrooke.ca/results/294/ (accessed on 20 November 2021).

Method	Basic	Interm.	Clutter	Jitter	Illumin.	Backgr.	Very	Very
		Motion			Changes	Motion	Long	Short
BB-SGD (ours)	**3.7881**	4.8898	**3.8776**	9.5374	4.5227	**8.5607**	5.6494	**4.1872**
SPMD [3]	3.8141	**4.1840**	4.5998	9.8095	**4.4750**	9.9115	6.0926	5.9017
LabGen-OF [4]	3.8421	4.6433	4.1821	9.2410	8.2200	10.0698	4.2856	5.0338
FSBE [5]	3.8960	5.3438	4.7660	10.3878	5.5089	10.5862	6.9832	5.4912
BEWIS [26]	4.0673	4.7798	10.6714	9.4156	5.9048	9.6776	**3.9652**	5.1937
Photomontage [14]	4.4856	7.1460	6.8195	10.1272	5.2668	12.0930	6.6446	4.9770
SOBS [28]	4.3598	6.2583	7.0590	10.0232	10.3591	10.7280	6.0638	5.2953
Temporal Median Filter [1]	3.8269	6.8003	12.5316	**9.0892**	12.2205	9.6479	6.9588	5.1336

**Table 3:** Evaluation results per criteria on the SBI dataset. ↓ indicates lower score is better, ↑ indicates higher score is better.

Method	Average	Average	Average	Average	Average
	AGE ↓	pEPs ↓	pCEPs ↓	MS-SSIM ↑	PSNR ↑
BB-SGD (ours)	**2.4644**	0.0083	0.0058	**0.9896**	**37.6227**
LabGen-OF [4]	2.7191	0.0145	0.0106	0.9824	35.9758
SS-SVD [24]	2.7479	0.0345	0.0907	0.9464	31.8116
LabGen [8]	2.9945	0.0139	0.0092	0.9764	35.2028
NExBI [15]	3.0547	**0.0077**	**0.0027**	0.9835	35.3078
BEWIS [26]	3.8665	0.0242	0.0142	0.9675	32.0143
Photomontage [14]	5.8238	0.0469	0.0372	0.9334	31.8573
SOBS [28]	3.5023	0.0415	0.0222	0.9765	35.2723
Temporal Median Filter [1]	10.3744	0.1340	0.1055	0.8533	28.0044

**Table 4:** AGE scores obtained using various truncated versions of the algorithm on 18 SBMnet sequences where a ground truth background is available.

Category	Video	Truncated Model				Full
		Version				Model
		v0	v1	v2	v3	
background motion
	advertisementBoard	1.61	1.62	1.60	1.34	1.71
basic
	511	3.42	3.44	3.43	3.44	3.43
	Blurred	1.80	1.69	1.68	1.68	1.61
clutter
	Foliage	32.87	5.86	3.62	3.41	3.37
	Board	21.37	6.78	7.84	7.37	7.39
	People and Foliage	31.36	9.66	3.75	2.54	2.60
	boulevardJam	21.37	15.89	19.5	11.0	2.03
illumination change
	CameraParameter	11.49	22.19	2.16	2.81	2.95
intermittent motion
	busStation	5.31	5.40	5.47	5.67	5.32
	Candela_m1.10	4.93	5.09	5.18	5.21	2.81
	CaVignal	12.57	12.61	13.58	14.04	2.05
	AVSS2007	10.98	10.32	10.25	10.01	8.73
jitter
	badminton	2.62	2.00	1.93	1.74	1.84
	boulevard	9.61	10.09	10.29	10.51	9.71
very long
	BusStopMorning	3.68	3.66	3.64	3.62	3.61
very short
	Toscana	8.79	8.80	8.79	3.30	3.30
	DynamicBackground	6.96	6.96	6.96	8.20	8.18
	CUHK_Square	2.77	2.77	2.77	2.99	2.98
Average AGE by category		8.06	7.53	4.94	4.51	3.75

**Table 5:** Impact of reducing the number of iterations on average AGE score and computation time.

Number of Iterations	100	250	500	1000	3000
Learning Rate	0.06	0.03	0.03	0.03	0.03
Computation time for 79 videos of the SBMnet dataset (seconds)	337	391	482	666	1409
Average AGE by category on 18 videos of the SBMnet dataset listed in Table 4	4.07	3.83	3.80	3.76	3.75
Average AGE on SBI dataset	2.78	2.56	2.53	2.49	2.46

## Data Availability

SBM.net dataset is available at the the following web address: http://scenebackgroundmodeling.net/ (accessed on 20 November 2021). The SBI dataset is available at the following web address: https://sbmi2015.na.icar.cnr.it/SBIdataset.html (accessed on 20 November 2021).
